# Advancements and future prospective of DNA barcodes in the herbal drug industry

**DOI:** 10.3389/fphar.2022.947512

**Published:** 2022-10-21

**Authors:** Karthikeyan Mahima, Koppala Narayana Sunil Kumar, Kanakarajan Vijayakumari Rakhesh, Parameswaran Sathiya Rajeswaran, Ashutosh Sharma, Ramalingam Sathishkumar

**Affiliations:** ^1^ Plant Genetic Engineering Laboratory, Department of Biotechnology, Bharathiar University, Coimbatore, Tamil Nadu, India; ^2^ Department of Pharmacognosy, Siddha Central Research Institute, Chennai, Tamil Nadu, India; ^3^ Department of Chemistry, Captain Srinivasa Murthy Central Ayurveda Research Institute, Chennai, Tamil Nadu, India; ^4^ Tecnologico de Monterrey, Centre of Bioengineering, Santiago de Queretaro, Queretaro, Mexico

**Keywords:** DNA barcoding, herbal products, monographs, quality control, regulatory status

## Abstract

**Ethnopharmacological relevance:** The past couple of decades have witnessed the global resurgence of medicinal plants in the field of herbal-based health care. Increased consumption of medicinal plants and their derivative products is the major cause of the adulteration issues in herbal industries. As a result, the quality of herbal products is affected by spurious and unauthorized raw materials. Recent development in molecular plant identification using DNA barcodes has become a robust methodology to identify and authenticate the adulterants in herbal samples. Hence, rapid and accurate identification of medicinal plants is the key to success for the herbal industry. Aim of the study: This paper provides a comprehensive review of the application of DNA barcoding and advanced technologies that have emerged over the past 10 years related to medicinal plant identification and authentication and the future prospects of this technology.

**Materials and methods:** Information on DNA barcodes was compiled from scientific databases (Google Scholar, Web of Science, SciFinder and PubMed). Additional information was obtained from books, Ph.D. thesis and MSc. Dissertations.

**Results:** Working out an appropriate DNA barcode for plants is challenging; the single locus-based DNA barcodes (*rbcL*, ITS, ITS2, *matK, rpoB, rpoC, trnH-psbA*) to multi-locus DNA barcodes have become the successful species-level identification among herbal plants. Additionally, multi-loci have become efficient in the authentication of herbal products. Emerging advances in DNA barcoding and related technologies such as next-generation sequencing, high-resolution melting curve analysis, meta barcodes and mini barcodes have paved the way for successful herbal plant/samples identification.

**Conclusion:** DNA barcoding needs to be employed together with other techniques to check and rationally and effectively quality control the herbal drugs. It is suggested that DNA barcoding techniques combined with metabolomics, transcriptomics, and proteomics could authenticate the herbal products. The invention of simple, cost-effective and improved DNA barcoding techniques to identify herbal drugs and their associated products of medicinal value in a fool-proof manner will be the future thrust of Pharmacopoeial monograph development for herbal drugs.

## Introduction

Medicinal plants and herbal supplements have contributed to a global resurgence in traditional health systems. Herbal medicines continue to gain international acceptance in modern medical and health care services. In India, traditional medical treatments such as Ayurveda, Naturopathy, Unani, Siddha and Homeopathy benefit humankind in a big way and are employed to treat diverse illnesses. Availability of genuine medicinal plants and its raw materials has increased in the past decade, testifying the worldwide interest in these products (Marichamy et al., 2014; Howard et al., 2020). Globalization of exporting the herbal medicines is expanding in the market leading to mixing of substitute materials or adulterants with genuine raw materials. Medicinal plants with high therapeutic potential are used for novel drug formulations in industries but the lack of standardized operating procedures and analytical methods, complicate the quality control of herbals. Herbal quality regulations vary between countries and authentication of herbal medicines relies on sensory and phytochemical screening techniques to detect species-specific characters and compounds respectively (European Medicine Agency, 2006; World Health Organization, 2004, (Word Health Organisation, 2011; EDQM, 2014). The substitution of unlabelled fillers used in the herbal medicines presents a challenge that risks patient safety and herbal efficacy. Several safety-related issues emerged globally due to the inaccurate or false identification of herbal medicines and their source plants. Therefore, the correct identification of herbal plants and their raw materials is essential for their safe usage.

Several traditional methods were used to authenticate herbal materials, including morphological, microscopic, and chemical identification. In the case of classical taxonomy approach involving the micro and macroscopic characters are not working recently due to the lack of taxonomic expertise available. Still, scientists have different opinions regarding the exact naming of species in the form of synonyms in classical taxonomy. The identification of the taxon is the fundamental activity and one of the primary objectives of plant systematics. It involves expert determination, recognition, comparison and the use of keys. The routine traditional identification decreases due to lack of taxonomist experts and often leads to misidentification among the closely related species.

Additionally, there was a lack of comprehensive morphological keys in different life stages of plants, phenotypic plasticity and genetic variability in the characters, which might significantly contribute to incorrect recognition and false identification of species (Vohra and Khera, 2013). Identification of taxa based on DNA sequence have the advantage that DNA sequence data is present uniformly in all plant parts and is relatively stable. In the last few decades, several genome-based techniques have been developed to identify plant species, but no single universally acceptable tool is known to identify plant species rapidly. Apparently, unique morphological characters and chemical constituents are found to be occasionally tough in distinguishing closely related species. The powdered or processed plant products cannot typically be identified without the help of pharmacognosy experts; however, like all techniques, they also have their own limitations. Pharmacognosy techniques offer herbal products quality and robustness with the involvement of trained experts, which eventually enable to separate the substitute from genuine samples (Li et al., 2011). An array of tools have been established, and each of them has its limitations in finding out the substitutions in the herbal samples (Kumar et al., 2009). The foremost techniques involve AFLP (Amplified Fragment Length Polymorphism) (Gowda et al., 2010), RFLP (Restriction Fragment Length Polymorphism) (Watthanachaiyingcharoen et al., 2010; Lin et al., 2012), CAPS (Cleaved Amplified Polymorphic Sequence), RAPD (Random Amplified Polymorphic DNA) (Hazarika et al., 2014), microsatellite markers or SSR (Simple Sequence Repeats) (Tamhankar et al., 2009), ISSR (Inter Simple Sequence Repeats) (Sharma et al., 2008), and SCOT (Start Codon Targeted Polymorphism) (Wang et al., 2001). By employing these techniques, multiple bands were observed with different sizes in an electrophoretic gel. Based on these qualitative data, different entities were identified and compared. However, the limitations of these techniques lie in the loss of specificity with the primer binding or restriction enzyme binding site. Another major drawback of studies employing the above-mentioned molecular techniques was the hardship of sequencing the multiple bands. In RAPD techniques, this was overcome with SCAR development (Sequence Characterized Amplified Region) markers. SCAR markers were achieved by sequencing a unique band for the species and developing primers from within. Even though SCAR markers help to identify the taxon at the species level, their function was rather suppressed with the determination of cultivars or varieties among the same species rather than classification among genus or familial level. So, there is a potential need to develop a genome-based approach for the exact identification of plant species (Sucher and Carles, 2008). The concept of “DNA barcoding” offers a comprehensive solution for many problems concerning plant identification. The emergence of DNA barcoding has positively impacted the herbal industry, biodiversity classification, and even the renaissance of taxonomy.

**FIGURE 1:**
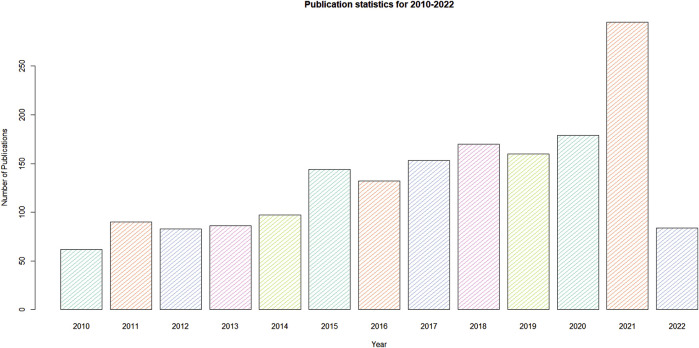
Graphical representation of manuscripts published from 2010 to 2022 on identification of medicinal plants using DNA barcoding.

DNA barcoding is a technique used to identify species based on a short-standardized portion of the genome. The three major principles of DNA barcoding are standardization, minimalism, and scalability. DNA barcoding can achieve rapid, time-saving and automated identification of species from all kinds of herbal products. By employing this technique, the extracted DNA from the collected sample using the standard protocol and following the DNA sequence analysis of the target gene harbors high rates of nuclear substitutions to discriminate closely related species while remaining more or less similar for all members of the same species. DNA barcoding was first put forward and universally accepted in animal systems (Hebert et al., 2003). However, in the case of plant species, individual taxons have not yet been discriminated due to the slow mutation rate. However, many studies have investigated universal plant barcodes, but no one has identified and discovered the ideal and universal barcode. Various regions of DNA showing high inter and intra-specific variability have been used as universal and high-resolution DNA barcodes. Two international initiatives were working to develop DNA barcodes in plants, including the Consortium for the Barcode of Life (CBOL) and the International Barcode of Life (iBOL). CBOL is a large group of scientists working intensively to identify DNA barcodes in flora and fauna globally. The iBOL is the largest biodiversity genomics group and their mission is to make DNA barcoding research as a global science. They maintained a cloud-based data storage platform named Barcode of Life Data systems (BOLD) reference library used for global species identification. It has been inferred that an ideal barcode region should have low intra-specific and high inter-specific divergence between the species (Kress and Erickson, 2007). However, there is controversy regarding the effective use of DNA barcodes in plants due to the poor discrimination ability among the species (Hebert et al., 2003; Moritz and Cicero, 2004; Will and Rubinoff, 2004; Ebach and Holdrege, 2005; Will et al., 2005; Newmaster et al., 2009). Several reports have convincingly shown that recognizing hidden diversity, monitoring biological invasions, finding out the different life stages of the seedlings, characterizing the molecular changes during metamorphosis, biodiversity monitoring, identification of fossil seeds, quality trade-in timber industries, monitoring the illegal trading of food products, identification of adulterants in commercial products including herbal supplements and assessment of diversified exotic species was possible with the employment of DNA barcoding techniques (Liu et al., 2011; Muellner et al., 2011; Baker et al., 2012; Gismondi et al., 2012). In CBOL (2009) recommended that single-locus plastid barcode-like *rbcL, matK* and two-locus combined *matK + rbcL* are the best plant barcode with high resolution and discriminatory power. The other regions of chloroplast and nuclear genome, such as *trnH-psbA, ycf1, ITS, trnL-F* and ITS2, have been recommended as supplementary DNA barcodes for plant identification (Hollingsworth et al., 2009; Ragupathy et al., 2009; Nithaniyal et al., 2014; Ferri et al., 2015). The application of NGS, whole-plastid genome and metabarcodes has stretched the versatility of DNA barcodes to the next higher level, proving the complete species information can be obtained irrespective of the morphological or life stages. The use of whole-plastid genome, mini-barcodes, and metabarcodes opened up a new way to identify plants (Erickson et al., 2008; Yang et al., 2012; Dormontt et al., 2018; Gao et al., 2019). However, the whole-plastid genome and mini-barcode concept have not been universally accepted due to difficulties obtaining the complete sequence and discrimination ability. However, metabarcoding is an emerging area of research with identifications of taxa from mixed samples by using the high throughput sequencing methods (Taberlet et al., 2012; Cristescu, 2014). DNA barcoding of medicinal plants and herbal products could be relatively challenging in evaluating the data to discriminate the exact species (Verma and Goswami, 2014). Figure 1 annotates the total number of international peer-reviewed manuscripts published from January 2010 to April 2022, denoting the use of DNA barcoding methods in medicinal plant identification. This review was revised to cover approximately 12 years till April 2022 publications of DNA barcoding papers related to medicinal plant and herbal products authentication from different platforms such as Google Scholar, Pubmed, Scopus and Web of Science. The current manuscript institutes the nature and amount of species adulteration reported in the herbal trade market and discusses the newly-developing techniques of DNA barcoding used to identify medicinal plants and, finally, insights into future research directions on DNA barcoding of medicinal plants and herbal medicines.

### Current status and role of regulatory authorities in herbal drugs

In recent decades, the demand for traditional medicines and herbal products increased exponentially in all over the globe and it led to the increment of investments in the sector. As the demands grows the quality of raw drug supplied got decremented and to compensate such issues World Health Organization (WHO) came up with various guidelines and regulatory strategies. The guidelines of WHO specifically mentioned the use of incorrect species is a threat to consumer safety (Palhares et al., 2015). Authenticating the quality of herbal medicines is one of the major regulatory requirement and countries over the globe came up with various regulatory organizations mitigate the challenge. Each country has its own Pharmacopoeia booklets of standards for drugs, pharmaceuticals, supplements *etc.* These books provide an acceptable quality monograph to maintain standards. The identification and authentication of raw materials using were traditionally carried out using organoleptic, morphological, microscopic characteristics and standard phytochemical assessments. DNA barcode-based authentication is now being applied in all industrial and pharmaceutical sectors to authenticate wide range of herbal raw drugs. In United States of America (USA), Food and Drug Administration (FDA) regulates the current Good Manufacturing Practices (cGMP) for dietary supplements (Palhares et al., 2015). In European Countries, European Medicines Agency (EMA) had guidelines concerning the quality of herbal drugs and products including qualitative and quantitative assays. Additionally, EMA encourage the use of other techniques to offer the assurance of quality in herbal medicines. The Canadian Food Inspection Agency (CFIA) also have the guidelines to authenticate the herbal drugs using the qualitative and quantitative assays (RSC, 1985). DNA barcodes have recently been incorporated into the British Pharmacopoeia for the first time (British Pharmacopoeia Commission, 2017). Recently, United States Pharmacopoeia, British Pharmacopoeia and Indian Pharmacopoeia have in recognition to test herbal drug authentication using ITS barcode candidate or other regions (Prakash et al., 2017). Currently, the Chinese Pharmacopeia have additions of Medicinal Materials DNA Barcode database (MMDBD) along with monographs (Wong et al., 2018). In India, many government organizations are working towards standardization, quality control and elimination of adulteration for herbal medicines. These include the Ministry of AYUSH, Central Council for Research in Ayurvedic Sciences (CCRAS), Central Council for Research in Siddha (CCRS), CSIR-Indian Institute of Integrative Medicine and Indian Pharmacopoeia Commission (IPC). The main objective of IPC is to develop comprehensive monographs about herbal drugs, which is in the form of an official book containing a detailed description of quality standards of herbal medicines, including raw herbs, herbal extracts, processed herbs and powdered ones, along with chemical information, preparation, function and regulation of drugs. However, well-compiled information on Indian herbal drugs with reference to Indian Pharmacopoeia is not yet available. To date total of eight IP editions have been published by the IPC (IPC, 2018). The current eighth edition of IP in 2018 consists of four volumes combining 220 monographs which include Chemical Monographs (170), Herbal Monographs (15), Blood and Blood-related products (10), Vaccines and Immunosera for Human use monographs (02), Radiopharmaceutical monographs (03), Biotechnology-Derived Therapeutic Products (06) and Veterinary monographs (14). The revised monographs are about 366 and seven commissions (IPC, 2018). The Indian Pharmacopoeia Commission has become the first WHO Collaborating Centre for Safety of Medicines and Vaccines in the South-East Asia Region (Basu, 2020). Indian Pharmacopoeia introduced DNA barcoding as a test to validate *Asparagus* species early in 2012, as alternative test when other test fails in species identification (Rai et al., 2012). This would ensure the wider use of herbal drugs at global level having regional to global markets and consumers across the globe.

### Unlabelled malpractices in the herbal industry

Despite various regulatory authorities and strict monitoring, certain malpractices persists on traditional drug trades. Traditional medical system and herbal products were linked with culture, economics, tourism and livelihood of regions, states or countries all around the globe. The industrial demand for medicinal plant resources makes a massive rise, with a matching proportion of adulterated herbal drugs sold out worldwide (Ved and Goraya, 2007). The primary reason for the supplementation of substitutes to authenticate samples is found to be mainly due to deforestation or extinction of many species and incorrect species identification (Mishra et al., 2015). If the demand for a specific herb is more remarkable, then there is an increased risk of adding adulterants and use of poor-quality materials. Another major malpractice in the herbal product industry arises due to the mismanagement of naming system. The traditional system follows vernacular names of plants over scientific names since majority of these system arise before the adaptation of scientific nomenclatures. Herbal formulation and ingredients were mentioned in ancient texts and manuscripts as in regional languages/vernacular names and the lack of experts in the identifying the plants based on these descriptions further escalates the situation. A well-known example was in the case of Brahmi; main ingredient in the production of memory enhanced drugs by Ayurveda and Siddha traditional system of medication. In northern states of India, the plant *Centalla asiatica* were considered as Brahmi while the southern states of India consider *Bacopa monneri* as Brahmi. This happens due to error in deciphering the identification features mentioned in traditional manuscripts and blindly following the vernacular naming of plant mentioned in various location. The plants *C. asiatica* and *B. monneri* comes under different families and have entirely different phytochemical composition and it form different effect in formulations (Santhosh Kumar et al., 2018). Further the traditional species identification is declining due to certain limitations such as lack of high-level taxonomic expertise, morphologically similar cryptic taxa and lack of incomplete morphological keys for particular life changes (de Boer et al., 2015). Another important factor contributing to the increase in malpractices in the herbal industry was the lack of proper regulation in distribution and selling of drugs. Unlike modern medicine traditional drug materials can be directly purchased from outlet without proper prescription from authenticated traditional medical practitioner. Purchasing of an improper drug based on vernacular naming and unwanted substitutions without proper prescription increase the chance of adverse health effect by multiple folds and which was one of the major causes of rejection towards traditional medicines in modern communities.

**FIGURE 2:**
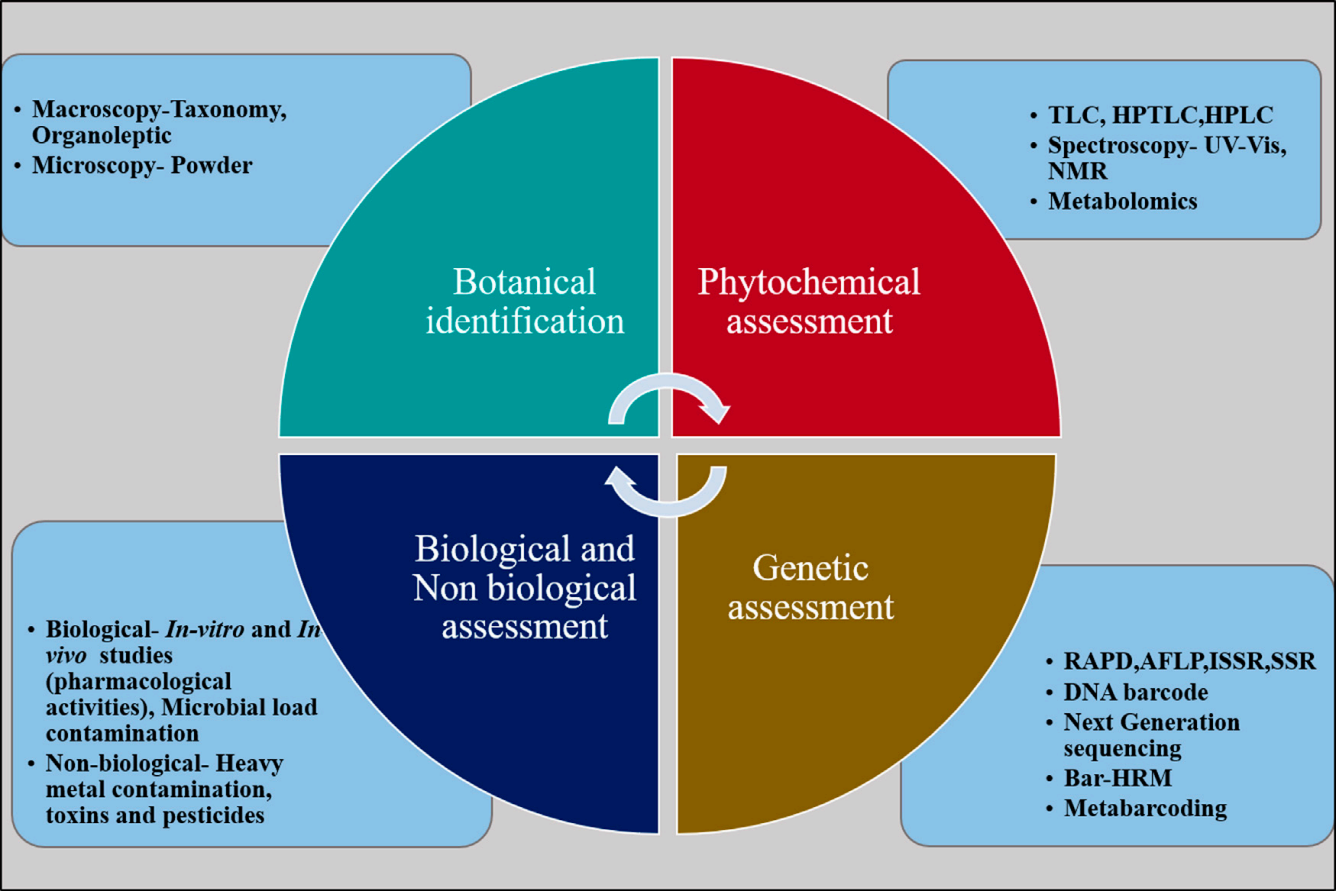
Parameters used in quality assessment of herbal products.

In addition to the adulteration with a substitute for the genuine biological species in herbal preparations, another problem contributing to falling quality standards of herbal products is related to the accumulation of heavy metals in the herbal-based drugs (Ernst, 2002; Chen et al., 2021). Saper et al., 2004 reported a significant amount of heavy metals in herbal products collected from Indian herbal samples (64% mercury, 41% arsenic and 9% cadmium). The traditional medicines from China, Mexico, Africa and South Asian countries have also been shown to contain heavy metals (Lekouch et al., 2001; Yu et al., 2021). These contaminants can lead to serious harm to patients such as, with problems associated with liver, kidney and respiratory leading to organ failure in the affected persons. There is a need for biological and chemical-based procedures in order to stringently evaluate the quality of herbal products. The quality control parameters for the evaluation of herbal products are summarized in Figure 2. In recent times new techniques have flourished to identify adulterants in the herbal samples, which are collectively termed as ‘Omics,’ a compilation of three technologies such as genomics, proteomics, and metabolomics (Pandey et al., 2016). Many reports based on wet-lab experiments showed that DNA barcoding technology could be used to find out the adulterants from herbal products and medicinal plants. For example, several reports discussed about the suitability of barcoding in ginseng species, a well-known group of medicinal plants (*Panax*, Araliaceae) and found that core barcodes *matK* and *rbcL* and additional ITS and *trnH-psbA* have been efficient for species identification (Zuo et al., 2011; Wallace et al., 2012). The studies on *Cassia* (Purushothaman et al., 2014), *Ginkgo* (Little, 2014), *Hypericum* (Howard et al., 2019), *Sida* (Vassou et al., 2015) and many other species have shown great utility in using DNA barcodes for the authentication of herbal products and medicinal plants. A few reports have employed modern techniques, such as microscopy, mass spectroscopy and metabolomics for the effective quality control of herbal products (Xiao et al., 2011; Raclariu et al., 2017b; Ichim et al., 2020). Based on the published resources with convincing experimental evidence, we tried our best to tabularize the list of adulterant species present in medicinal plants (Table 1).

**TABLE 1:** List of known adulterants/substitutes in the herbal drug trade.

S.No	Main drugs	Adulterants	References
1	*Aconitum heterophyllum*	*Cyperus rotundus*	Seethapathy et al. (2014)
2	*Aerva lanata*	*Aerva javanica, Bergenia ligualata*	Sarin, (1996)
3	*Aloe barbadensis*	*Acacia catechu*	Keshari and Pradeep, (2017)
4	*Amomum subulatum*	*Heracleum rigens*	Keshari and Pradeep, (2017)
5	*Andrographis paniculata*	*Swertia chirayta*	Mishra et al. (2015)
6	*Asparagus racemosus*	*Asparagus gonoclados*	Rai et al. (2012)
7	*Atropa belladonna*	*Ailanthus altissima*	Ahmed and Hasan, (2015)
8	*Bacopa monnieri*	*Centella asiatica*	Santhosh Kumar et al. (2018)
9	*Bereberis aristata*	*Coscinium fenestratum*	Santhosh Kumar et al. (2018)
10	*Boerhavia diffusa*	*Boerhavia erecta, Boerhavia repanda, Boerhavia coccinea, Boerhavia verticillata*	Selvaraj et al. (2012)
11	*Cassia fistula*	*Senna auriculata*	Seethapathy et al. (2015)
12	*Cassia angustifolia*	*Cassia obtusifolia*	Sultana et al. (2012)
13	*Cinnamomum obtusifolium*	*Cinnamomum tamala*	Dhanya and Sasikumar, (2010)
14	*Cinnamomum zeylanicum*	*Cinnamomum cassia Cinnamomum malabatrum*	Swetha et al. (2014)
15	*Cinnamomum verum*	*Canella winterana Cinnamomum malabatrum*	Swetha et al. (2014)
16	*Citrullus colocynthis*	*Trichosanthes palmata*	Osathanunkul et al. (2016)
17	*Cuscutare flexa*	*Cuscuta chinensis*	Khan et al. (2010)
18	*Commiphora wightii*	*Boswellia serrata, Hymenodictyon excelsura*	Prakash et al. (2013)
19	*Commiphora mukal*	*Acacia nilatica, Commiphora myrrha*	Ahmed et al. (2011)
20	*Convolvulus microphyllus*	*Evolvulus alsinoides*	Prakash et al. (2013)
21	*Crocus sativus*	*Carthamus tinctorius, Zea mays, Calendula offinalis, Curcuma longa, Nelumbo nucifera*	Jiang et al. (2014)
22	*Emblica ribes*	*Emblica robusta, Embelia tsjeriam-cottam, Maesa indica*	Santhosh Kumar et al. (2018)
23	*Ferula foetida*	*Acacia senegal*	Prakash et al. (2013)
24	*Gloriosa superba*	*Ipomea spp.*	Sagar, (2014)
25	*Glycyrrhiza glabra*	*Glycyrrhiza uralensis; Abrus precatorius*	Khan et al. (2010)
26	*Habenaria edgeworthii*	*Dioscorea bulbifera*	Prakash et al. (2013)
27	*Hemidesmus indicus*	*Decalepis hamiltonii*	(Rai et al., 2012; Kesanakurti et al., 2020)
28	*Holarrhena antidysenterica*	*Wrightia tinctoria, Wrightia tomentosa*	Gahlaut et al. (2013)
29	*Hypericum perforatum*	*Hypericum patulum, Hypericum maculatum*	Raclariu et al. (2017b)
30	*Justicia adhatoda*	*Ailanthus excelsa*	Keshari and Pradeep, (2017)
31	*Mesua ferrea*	*Calophyllum inophyllum*	Keshari and Pradeep, (2017)
32	*Morinda citrifolia*	*Moringa oleifera*	Santhosh Kumar et al. (2018)
33	*Mucuna pruriens*	*Mucuna utilis, Mucuna deeringiana*	Vijayambika et al. (2011)
34	*Myristica fragans*	*Myristica malabarica*	Swetha et al. (2017)
35	*Ocimum tenuiflorum*	*Ocimum basilicum*	Kumar et al. (2020)
36	*Papavar somniferum*	*Amaranthus paniculatus*	Dhanya and Sasikumar, (2010)
37	*Paris polyphylla*	*Acorus calamus*	Arya et al. (2012)
38	*Parmelia perlata*	*Parmelia perforate, Parmelia cirrhata*	Ahmed and Hasan, (2015)
39	*Penthorum sedoides*	*Penthorum chinese*	Duan et al. (2011)
40	*Phyllanthus amarus*	*Phyllanthus debilis, P. urinaria*	Srirama et al. (2010)
41	*Piper kadsura*	*Piper wallichii, piper boehmeriafolium, Piper laetispicum*	Yu et al. (2016)
42	*Piper longum*	*Piper mullesua*	Mishra et al. (2015)
43	*Piper nigrum*	*Capsicum annum, Carica pappaya*	(Parvathy et al., 2014; Mishra et al., 2015)
44	*Plumbago auriculata*	*Plumbago zeylanica*	Keshari and Pradeep, (2017)
45	*Pterocarpus santalinus*	*Capsicum annum*	Keshari and Pradeep, (2017)
46	*Rubia cordifolia* L	*Rubia tinctorum*	Kamani et al. (2021)
47	*Ruta graveolens*	*Euphorbia dracunculoides*	Al-Qurainy et al. (2011)
48	*Santalum album*	*Erythroxylum monogynum*	Keshari and Pradeep, (2017)
49	*Saraca asoca*	*Polyalthia longifolia, Humboldtia vahliana, Mallotus nudiflorus*	(Beena and Radhakrishnan, 2012; Begum et al., 2014)
50	*Saussurea lappa*	*Saussurea costus*	Sagar, (2014)
51	*Sida cordifolia*	*Sida acuta*	Ran et al. (2010)
52	*Solanum nigrum*	*Solanum melogena*	Prakash et al. (2013)
53	*Swertia chirata*	*Swertia augustifolia*	Osathanunkul et al. (2016)
54	*Terminalia arjuna*	*Terminalia tomentosa, Terminalia bellirica., Terminalia chebula*	Keshari and Pradeep, (2017)
55	*Tinospora cordifolia*	*Tinospora sinensis*	Keshari and Pradeep, (2017)
56	*Tribulus terrestris*	*Tribulus lanuginosus, Tribulus subramanyamii*	Balasubramani et al. (2010)
57	*Ventilago madraspatana*	*Arnebia euchroma*	Ahmed and Hasan, (2015)
58	*Withania somnifera*	*Mucuna pruriens, Trigonella foenum-graceum, Senna auriculata*	Amritha et al. (2020)
59	*Zingiber officinale*	*Zingiber mioga*	Ahmed and Hasan, (2015)

## DNA barcoding: A genomics-based tool for plant identification

Since its initiation in 2003, DNA barcoding has drawn the attention of the international scientific community, government agencies and the public. Parallel development in the field of biotechnology and plant taxonomy creates a rejuvenated emphasis on the exploration and rapid identification of species. Hebert et al., 2003 proposed a microgenomic identification system or DNA taxonomy, which permits life discrimination by analyzing a small standardized genome segment. This represents one of the promising approaches towards diagnosing biological diversity. It implies that the standard DNA locus is amenable to bidirectional sequencing, which effectively provides high discrimination among the species. These innovations contribute to major advancement in the plant systematics classification and identification of taxa of medicinal importance (Kress et al., 2005). DNA barcoding has become a reality in recent times, and various markers have been used with reference to its universality and high resolution between the species (Chen et al., 2010, 2014). Intended for the discrimination, DNA markers need to be in higher inter and lower intraspecific divergence, called ‘DNA barcoding gap.’ For several years, CBOL and many studies have searched for emphasized an efficient and universal plant barcode; it was inferred that none of the available loci could work across all species (Chase and Fay, 2009; Chen et al., 2010). The Consortium for Barcode of Life-Plant Working Group (CBOL) recommended the chloroplast (*matK, rbcL*) and combination of *matK + rbcL* are to be the ideal barcodes for all the plant species (Kress et al., 2005; Rubinoff et al., 2006; CBOL, 2009; Stoeckle et al., 2011). Recently it has been proposed that by employing the molecular attributes of the whole-plastid genome in plant identification, flawless identification of herbal plants could be achieved. However, this concept and approach have has not yet been accepted universally (Erickson et al., 2008; Sucher and Carles, 2008; Yang et al., 2012; Ahmed, 2022). One of the main reasons is found to be the high sequencing cost and the difficulties in obtaining a complete plastid genome as compared to single-locus barcodes. In many of the taxa, secondary metabolites which are present in the leaves, stems and roots often hamper successful PCR conditions and these shortcomings are known to be overcome by making suitable modifications in the extraction methods. The types of DNA barcode markers and the related techniques have been discussed in the following passages, emphasizing the current trends for overcoming the challenges of DNA barcoding in plants.

### Single-locus DNA barcode markers

DNA barcoding studies primarily aim to develop a universal DNA barcode marker in plants to identify taxa. Many reports have recommended developing a universal barcode marker from the plastid and nuclear genome (Newmaster et al., 2006; Kress and Erickson, 2007; Kress, 2017). In 2009, Centre Barcode for Life Plant Working Group compared seven barcode candidates, which namely *rbcL, matK, rpoC1, rpoB, trnH-psbA, psbK-psbI* and atpF-atpH) distributed in 550 plant species and suggested that *matK and rbcL* markers could serve as the core barcode candidates for plant species identification and indicated that ITS and *trnH-psbA* could be employed as supplementary markers. In Chen et al. (2010) worked on various medicinal plants and herbal materials from more than 6,600 samples in 753 genera and proposed that ITS2 could serve as a core DNA barcode for medicinal plants and herbal materials. In addition, the chloroplast marker *trnH-psbA* has been proposed as a complementary barcode marker. Both ITS2 and *trnH-psbA* are known to be shorter genes with conserved sequences, which reduces the difficulty of amplification. Chen et al. (2010) in a series of publications, reported that the efficiency of ITS2 and *trnH-psbA* markers aided easy identification of medicinal and herbal materials (Chen et al., 2013, 2014). Since then, many studies have examined several gene regions which helped in the identification of species, including *accD* (He et al., 2014a; Mao et al., 2014), *atpF-atpH* (Ran et al., 2010; Zuo et al., 2011; Thakur et al., 2019, 2021), *rpoB* (Zuo et al., 2011; Singh et al., 2012; Liu et al., 2016), *ndhJ* (He et al., 2014a; 2014b), *ycf1* and *ycf5* (Luo et al., 2010; Dong et al., 2015), *rpoC1* (Luo et al., 2010; Shi et al., 2011; Khan et al., 2012; Singh et al., 2012; Aziz et al., 2015; Naim and Mahboob, 2020).

### Multiple-locus DNA barcode markers

It has been shown that single-loci marker cannot always be authenticated for species identification; accordingly, scientists have used a combination of DNA markers. The plant working group CBOL recommended the combined loci of *matK* and *rbcL* as the core barcode for plant species (Fazekas et al., 2008; Newmaster et al., 2008; CBOL, 2009). Additional combinations of markers, including *rbcL + trnH-psbA, trnH-psbA* + ITS2, and *matK + trnH-psbA*, have also been assessed for their efficiency in discrimination and universality of species identification (Kress et al., 2005; Kress and Erickson, 2007; Li et al., 2012; Tripathi et al., 2013; Mishra et al., 2015; Mahima et al., 2020). Evaluation of the reports related to multiple loci DNA in plant identification revealed that, the combined loci of ITS2 and *trnH-psbA* were found to be the best two-marker combination for the identification of plants and even herbal samples (Zuo et al., 2011; Chen et al., 2014; Vassou et al., 2015; Jamdade et al., 2022). By employing the combined barcode loci a deep phylogenetic tree can be established, which could be applied in the identification of species and also discrimination of closely related species. In addition, the combination of a third locus is also reported for the large datasets but it was observed to have low bias at the species level (Newmaster et al., 2013). The list of successful resolution of DNA barcode for family-level identification are mentioned in Table 2.

**TABLE 2:** List of recommended DNA barcode loci for medicinal plant families.

S.No	Family	Best locus/Loci	References
1	Asteraceae	ITS	Gao et al. (2010)
2	Apiaceae	ITS/ITS2 + *psbA-trnH*, ITS, ITS2	(Liu et al., 2014; Parveen et al., 2019)
3	Apocynaceae	ITS2+*trnH-psbA, matK + rbcL*	(Cabelin and Alejandro, 2016; Lv et al., 2020)
4	Araliaceae	ITS2	Liu et al. (2012)
5	Arecaceae	*rbcL*	Naeem et al. (2014)
6	Euphorbiaceae	ITS1, ITS2	Pang et al. (2011)
7	Fabaceae	ITS2	Tahir et al. (2018)
8	Lamiaceae	*matK, rbcL*	Oyebanji et al. (2020)
9	Lauraceae	ITS	Liu et al. (2022)
10	Lemnaceae	*atpF-atpH*	Wang et al. (2010)
11	Meliaceae	ITS	Muellner et al. (2011)
12	Myristicaceae	*matK + trnH-psbA*	Newmaster et al. (2008)
13	Orchidaceae	*ndhF, ycf1, matK + ycf, ndhF + ycf1*	Li et al. (2021)
14	Poaceae	ITS1, ITS2	(Yao et al., 2017; Tahir et al., 2018)
15	Polygonaceae	*trnH-psbA*	Song et al. (2009)
16	Rosaceae	ITS2	Pang et al. (2011)
17	Ranunculaceae	*rbcL + matK + trnH-psbA*, ITS	Li et al. (2019)
18	Rutaceae	ITS2	Luo et al. (2010)
19	Verbanaceae	*matK, rbcL*	Oyebanji et al. (2020)
20	Zingiberaceae	ITS2, *rbcL, matK*	Vinitha et al. (2014)

### High-throughput sequencing technologies

In the last decade, many scientists have employed single or multi-locus of DNA barcode markers in order to identify species distributed across different families, genera and species of medicinal plants. Further, the emergence of Next-generation sequencing made a revolution in the extensive analysis of mitochondrial, chloroplast and nuclear genomes of many organisms. The high-throughput technology platform generates a vast amount of sequence data in a matter of hours or days, with an algorithm to precisely address the needs of each application. The technique involves the initial fragmentation of DNA templates followed by the immobilization of the fragments on a solid support. Subsequently, the fragments are amplified and sequenced. Three distinct strategies are being employed in practicing NGS and this technology has been commercialized by Roche’s 454 Life Science platform (Indianapolis, IN), Illumina/Solexa Genome Analyzer (San Diego, CA) and Applied Biosystems/SOLiD System (Orange countery, CA). Each strategy has its unique enzyme systems, sequencing chemistry, hardware and software systems (Shendure and Ji, 2008; Metzker, 2010). Among all, Roche/454 is found to be more advantageous due to its rapid and longer read length with a hundred thousand to one million reads of 400–500 bp DNA fragments per run (Sarwat and Yamdagni, 2016), even while performing the technique in the identification of plant species. In the routine analysis, the entire genome has been employed for phylogenetics, especially at a deeper level, genome evolution analysis and authenticating medicinal plants for herbal drug preparations (Kircher and Kelso, 2010; Ganie et al., 2015; Sarwat and Yamdagni, 2016). In-plant species, chloroplast genomes are preferred for species identification which contains more information. By July 2021, the chloroplast genome of plants has been published on NCBI. Many plant scientists suggested that the entire plastid genome could be a powerful tool to resolve the phylogenetic relationship between closely related species, identification of the homogeneity of samples and the presence of adulterants in herbal supplements (Li et al., 2015; Zhou et al., 2018). Several reports have been found that Next-generation Sequencing (NGS) technique for the authentication and quality control of herbal medicines (Zhang et al., 2017). Cheng et al. (2014) accomplished a metagenomic analysis of Liuwei Dihuang Wan herbal medicine to find the biological ingredients and contaminations involved. The results showed that quality and stability of different manufacturers are significantly dissimilar. In addition, Speranskaya et al. (2018) performed two sequencing platforms, Illumina and Ion Torrent to identify high-quality qualitative and quantitative results. The whole plastid sequences of 57 *Berberis* species were determined to identify the informative DNA barcodes and understand the phylogeny between species (Kreuzer et al., 2019). In the context of quality control of herbal medicines, Nanopore sequencing has been used for herbal product authentication (Lo and Shaw, 2019).

### DNA barcoding technique combined with other technologies

After more than 15 years of development, DNA barcoding has been employed to rapidly identify species using standardized genetic markers (Hebert et al., 2003). This technique has been successfully applied in raw plant herbal extracts and has shown to have a few limitations in herbal products subjected to heating, purification and leaching, which results in DNA degradation and makes the extraction difficult (Govindaraghavan et al., 2012; Liu et al., 2016; Raclariu et al., 2018). The quality of DNA is known to play an essential role in the authentication of medicinal plants and herbal products. For this purpose, different techniques have been employed to overcome the limitation in performing DNA barcoding, such as DNA mini-barcoding, Bar-HRM technology and Metabarcoding. Additionally, there are a few analytical methods such as, chromatography that has been combined with barcode markers, including TLC (Thin Layer Chromatography), HP-TLC (High Performance-Thin Layer Chromatography), HPLC (High-Performance Liquid Chromatography) and LC-MS (Liquid Chromatography-Mass Spectrometry). These methods have been shown to be highly useful in identifying the active components of medicinal plants and herbal products (Palhares et al., 2015). The first report on Salvia species showed the relationships between the DNA barcoding technique and chemical components (Jianping et al., 2010). The authentication of *Hypericum* from the herbal products using TLC and HPLC combined with Amplicon Metabarcoding (AMB) (Raclariu et al., 2017b). Zhao et al. (2020) demonstrated a systematic method to authenticate *Stephania* species which involved a combination of DNA barcoding, HPLC-QTOF-MS/MS and UHPLC for differentiation, chemical profiles and quality evaluation. NMR (Nuclear Magnetic Resonance) and genome skimming were recently combined to validate *Hemidesmus indicus* from the adulterant species *Decalepis hamiltonii* on both raw materials and finished products. This was the first report on the use of Oxford Nanopore on herbal products enabling genome skimming as a tool for quality assurance perspective for both product and purity (Kesanakurti et al., 2020).

### DNA mini-barcoding

DNA mini-barcoding is a complementary technique to DNA barcoding. These mini barcodes were used mainly in discriminating species in a genus. It was a small, highly conserved portion in the usual barcode region, and specialized primers were synthesized to target the area. The main advantage of doing mini barcodes was the ease of sequencing of less than 200 bp and used to make the comparison within the species. Meusnier et al. (2008) demonstrated that DNA-mini barcodes could overcome the difficulties associated with degraded samples of processed herbal products. Recently, ITS2 has been reported as a suitable mini-barcode for identifying medicinal and adulterant species from the family Apiaceae (Parveen et al., 2019). These mini-barcodes are limited by length restrictions and sequences of different lengths are selected as mini-barcodes. The success of mini-barcodes are dependent on specific primers which need to be screened from the available databases such as GenBank, the European Molecular Biology Laboratory or the DNA Data bank from Japan (Hajibabaei et al., 2007; Gao et al., 2019).

#### Bar-HRM technique

The Bar-HRM technique is based on DNA barcoding coupled with high-resolution melting analysis. The melting curve of PCR amplicons is subject to length of DNA sequence, GC content and difference in base complementary without sequencing or hybridization procedures; thus, *rbcL, matK, trnH-psbA, rpoC* and ITS, *etc.* can be employed to facilitate species identification (Jiang et al., 2014; Yu et al., 2021). HRM monitors the melting curve of the nucleic acid in real-time by adding intercalating dyes, including SYBR Green, Green PLUS, Eva Green, SYTO9 and ResoLight. This new approach has the distinct advantage of performing both PCR amplification and HRM analysis in one completely ‘closed tube’ and results are available at the end of the run. These advantages make it widely used in the herbal medicine industry and market to determine the origin and quality of raw materials and detect the adulterants in herbal processed products (Sun et al., 2016; Mezzasalma et al., 2017). In addition, HRM analysis is used for clinical research and diagnostics, including the analysis of cancer-specific mutations (Li et al., 2016), authentication of food products (Ganopoulos et al., 2011; Madesis et al., 2013; Sakaridis et al., 2013) and detection of harmful microorganisms in meat products (Sakaridis et al., 2014; Ganopoulos et al., 2015). An overview of the Bar-HRM analysis developed in herbal medicine identification is provided in Table 3. In first reported the advantages in applying and utilizing the HRM approach for the rapid discrimination of seven Greek *Sideritis* species, which was carried out based on nuclear ITS2 DNA barcoding sequence. Since then, another research group has approached plastid DNA region *trnH-psbA* coupled with HRM analysis to distinguish Chinese medicine *Panax notoginseng* from adulterant species (Tong et al., 2014), and the same method was used for the identification of saffron (*Crocus sativus*), Mutong (*Akebia quinata*) and Aristolochia (*Aristolochia manshuriensis*) (Jiang et al., 2014; Hu et al., 2015). These few studies revealed that the HRM analysis could distinguish original species from adulterant species. However, the drawback in the Bar-HRM analysis is the failure in the identification of closely related species where genetic variability is limited. To overcome the issue, (Osathanunkul et al., 2015b), developed a specific mini-barcode in the *rbcL* gene to distinguish three medicinal plants in the Acanthaceae (*Acanthus ebracteatus*, *Andrographis paniculata* and *Rhinacanthus nasutus*). Singtonat and Osathanunkul, (2015) reported that *Thunbergia laurifolia* derived herbal products could be identified by employing four DNA mini barcodes: *matK, rbcL, trnL* and *rpoC*. In addition, mini-barcode coupled with HRM analysis was able to detect the toxic herb *Crotalaria* *spectabilis* adulterants. *Lavandula* species was successfully authenticated using Bar-HRM technology (Soares et al., 2018). The narcotic plant, *Mitragyna speciosa* has morphological disparities with allied *Mitragyna speciosa* and the results showed that the melting profiles of ITS2 amplicons were distinct from allied species (Tungphatthong et al., 2021). Zhao et al. (2021) demonstrated that DNA barcoding coupled with a high-resolution melting curve could be used as a routine test to guarantee the quality of *Ardisia gigantifolia* and to discriminate the genuine species from its common adulterants.

**TABLE 3:** List of recommended barcode locus/loci for the application of Bar-HRM in medicinal plant identification.

S.No	Description	DNA barcode region	References
1	*Sideritis species*	ITS2	Kalivas et al. (2014)
2	*Panax notoginseng*	*trnH-psbA*	Tong et al. (2014)
3	*Aristolochia manshuriensis*	*trnH-psbA*	Hu et al. (2015)
4	*Acanthus ebracteatus*, *Andrographis paniculata*, and *Rhinacanthus nasutus*	*rbcL*	Osathanunkul et al. (2015a)
5	*Hypericum perforatum* and *Hypericum androsaemum*	ITS1 and *matK*	Costa et al. (2016)
6	*Phyllanthus amarus*	*trnL* and *rbcL*	Buddhachat et al. (2015)
7	Croton species	ITS1, *matK, rbcL, rpoC* and *trnL*	Osathanunkul et al. (2015b)

### Metabarcoding

Another approach merged from DNA barcoding which has been advocated for identifying taxa from a complex mixture, called ‘metabarcoding.’ It combines both DNA barcoding and high-throughput sequencing. In the recent times, significant methodological advancements have taken place in high-throughput sequencing along with the employment of bioinformatics tools in order to obtain amplified sequences to identify species diversity from the environment, sediment, and ancient or processed samples (Taberlet et al., 2012; Omelchenko et al., 2019). It is inferred that microbial flora determination was the target for metabarcoding. Additionally, metabarcoding can be implemented to identify multiple plant species as well as processed herbal products using the universal primers (Cheng et al., 2014; Ivanova et al., 2016; Raclariu et al., 2017a, 2017b). For example, Coghlan et al. (2012) demonstrated metabarcoding studies on 15 complex traditional Chinese medicine samples and identified 68 families, including possible toxic species. In Ivanova et al. (2016) tested 15 herbal supplements from the market and found non-listed non-filler plant DNA. Out of 78 *Hypericum perforatum* samples in the herbal market revealed that 68% of samples are authenticated and the rest are adulterants (Raclariu et al., 2017b).

Raclariu et al. (2017a) investigated *Veronica officinalis* and found out that adulterant species *Veronica chamaedrys* could be detected in 62% of the products. Additionally, the same group found adulterant species in 53 *Echinacea* herbal products (Raclariu et al., 2018a; 2018b). Assessment of 79 Ayurvedic herbal products on the European market using DNA metabarcoding analysis revealed that two out of 12 single-ingredient products contained only one species as labeled and from the 27 multiple ingredient products, only eight species could be authenticated and none of the species are not listed on the label. The study highlights that DNA metabarcoding is an appropriate analytical approach for authenticating complex multi-ingredient herbal products (Seethapathy et al., 2019). Recently, Urumarudappa et al. (2020) identified 39 Thai herbal products on the Thai National List of Essential Medicines (NLEM) which revealed that the nuclear region, ITS2, could identify herbal ingredients at the genus and family level as 55% and 63%, respectively. The chloroplast gene, rbcL, is known to enable genus and family level identification in 58% and 73% of cases. The study recommended that advanced chemical techniques combined with DNA metabarcoding could be valid for multi-ingredient herbal products. Moreover, a specific mini-barcode is coupled with DNA metabarcoding technique used for the qualitative and quantitative identification of *Senna* processed herbal products (Yu et al., 2020). All these studies showed varying degree of authentication success since the complex herbal mixtures is known to be influenced by several factors, which also includes the quality and type of material as well as several analytical parameters and variables that are employed in the process of optimization of experimental results (Staats et al., 2016).

There are also a few other limitations of DNA metabarcoding especially if the DNA has been degraded or lost during the manufacturing process of herbal products (de Boer et al., 2015). Generally, with the employment of metabarcoding, one can obtain accurate and reliable high-quality sequences to identify the species through high throughput sequencing within complex multi-ingredient and processed mixtures (Veldman et al., 2014; Jia et al., 2017). Importantly, this technique is useful for qualitative evaluation and not suitable for quantitative assessment, especially in evaluating relative species abundance based on sequence read numbers (Coghlan et al., 2012; Staats et al., 2016). In addition, it can be ascertained that the third-generation sequencing PacBio platform combined with DNA barcoding is bound to make a greater impact on authenticating herbal products in the future (Senapati et al., 2022).

### Loop-Mediated Isothermal Amplification (LAMP) and Recombinase Polymerase Amplification (RPA)

Several molecular biology methods are available for the identification of herbal medicines, and the advantages over other methods are rapidity, high sensitivity, and specificity. As an alternative to a Polymerase Chain Reaction (PCR), Loop-Mediated Isothermal Amplification (LAMP) and Recombinase Polymerase Amplification (RPA) were introduced into herbal medicines for safety testing. LAMP can be implemented with impure sample materials as the template and has a short reaction time, and does not require specific equipment. The application of LAMP has exhibited great potential in the field of herbal medicine identification. LAMP amplification is performed with accessible primers to crosscheck their species-specific identification. Species identification is determined by analyzing the turbidity curve along with visual colour changes instead of the agarose gel DNA electrophoresis test. Recent studies proved that the implementation of LAMP analysis is effective in herbal medicine identification. The first report of the LAMP-based method is to discriminate the identity of *Curcuma longa* and *C. aromatica* by targeting the *trnK* gene sequence. The results showed that LAMP analysis is suitable for the identification of herbal medicines (Sasaki and Nagumo, 2007). In the following year, targeting six allele-specific markers (18S ribosomal RNA gene) is used for the detection of *Panax ginseng* from *Panax japonicus* (Sasaki et al., 2008). The identification of traditional Chinese medicine *Cordyceps sinensis* from its adulterant *C. hawresii, C. ramosa, C. militaris*, and *C. barnesi* through the same approach. In the other study, the LAMP method is combined with RAPD to identify *Catharanthus roseus* and the results showed high specificity (Chaudhary et al., 2012). Li et al. (2013) demonstrated the LAMP method targeting internal transcribed spacer (ITS) for the authentication of herbal tea ingredient *Hedyotis diffusa*. Lai et al. (2015) developed a similar approach and evaluated the effectiveness of the ITS2 DNA barcode in differentiating *Taraxacum* *formosanum* from its adulterants. In 2016, Zhao et al., 2016. Developed a species-specific primer for *Crocus sativus* from its adulterants through LAMP analysis. These studies suggested that the advent of LAMP based specific primers effectively identify medicinal plant species from their non-medicinal adulterants.

Recombinase polymerase amplification (RPA) is a unique isothermal DNA amplification developed by Pipenburg et al. (2006). The DNA barcode-based RPA (BAR-RPA) technique requires recombinase, polymerase, and single-stranded binding protein (SBB) to replace the unwinding chain process of the usual PCR technique. Recombinase polymerase amplification allows rapid amplification (approximately 20 min) of genetic markers under a constant temperature of 35–40°C with or without the use of thermocyclers and coupled with a rapid DNA extraction method (Lobato and O’Sullivan, 2018). The resulting RPA reaction products can be visualized in agarose gel electrophoresis, probe-based fluorescence monitoring, and lateral flow dipstick (LFD). In Tian et al. (2017) reported a reliable protocol of DNA extraction and combine it with RPA-LFD to establish a rapid authentication of *Ficus hirta* from its adulterant, a toxic plant *Gelsemium elegans*. There are few reports that documented the use of the RPA technique for the identification of herbal medicines and their adulterants. This technique can provide an effective detection in the finding of adulteration, forensic medicine, and molecular assays. In addition, such applications of this technology can help to improve the safety of herbal medicines, especially combined with authentication *via* morphological, chemical, or other molecular methods.

### Current databases

Establishing a database is essential for identifying and authenticating all flora and fauna, keeping the information updated and organized, and making the sequence data accessible to all scientific communities worldwide. The aim of databases is to collect, manage and analyze the sequence data from the diverse organism and the most popular databases are 1) National Center for Biotechnology Information (NCBI) GenBank, 2) BOLD Systems (Barcode of Life Data Systems) and 3) MMDBD (Medicinal Materials DNA Barcode Database). Currently, MMDBD is the only database having the sequence data of medicinal plants listed in Chinese Pharmacopoeia (Wong et al., 2018).

NCBI GenBank (http:/www.ncbi.nlm.nih.gov/genbank) is an online database that contains the publicly available genetic information of prokaryotes and eukaryotes organisms. This database has a vast sequence of DNA, RNA and proteins. The unknown species could be identified through the BLAST (Basic Local Alignment Search Tool) algorithm. The species with highest similarities are present at the first-come position and an E value ≥ 0. Unlike the BOLD database, NCBI GenBank does not maintain the chromatogram of the sequences submitted to the platform.

The BOLD (http://www.boldsysystem.org) is a virtual platform for all eukaryotes organisms and it is hosted by the University of Guelph in Ontario, Canada. This bioinformatics workbench assists in analyzing, storing, and publishing DNA barcode records and chromatograms. The user can directly submit their data without any significant difficulties and give each species a unique accession ID. BOLD accepts the sequence from more than 150 genetic markers, including *COI*, ITS, *rbcL* and *matK*. These records include sequence data, barcodes, images, taxonomy, maps, and collection coordinates data. The barcode sequence of unknown species can be rapidly and accurately identified using the online database to support their identification/validation. Besides, this online platform helps collaborate between geographically dispersed research communities with web-based delivery. For example, Vassou et al. (2016) retrieved the 27 medicinal plants *rbcL* sequence from the BOLD public database to create the Ayurvedic Pharmacopoeia of India-Reference DNA Barcode Library (API-RDBL). In addition, the sequence generated for the study were deposited in the BOLD database. Similarly, Gong et al. (2018) constructed the first local reference barcode library for Southern Chinese Medicine using the ITS2 sequence. The partners in the BOLD database are iBOL [[International Barcode of Life (http://www.ibol.org)], CBOL [Consortium for the Barcode of Life (http://www.barcodeoflife.org)] and GBIF [Global Biodiversity Information Facility]. The CBOL is a public online database containing many DNA barcode sequences to identify unknown species. This database originated in 2004 and the founders promote the scientific community to conduct the DNA barcoding conference, meetings, training and classes to reach public support. Currently, its works with 130 organizations in 40 different countries. Their mission is to collect and record all sequence data from eukaryotes worldwide and make it available to public reach. iBOL was established in 2008 and it aims to generate DNA barcode libraries to identify the biodiversity with a standard protocol and bioinformatics tools. iBOL has collaborated with the BIOSCAN program to achieve 2.5 million species with barcodes by 2025.

MMDBD (https://rdccm.cuhk.edu.hk/mherbsdb) is an online platform established in 2010 and that can be used for DNA sequence identification and data retrieval. This platform conta ins the sequence of medicinal plants listed in Chinese Pharmacopoeia and American Herbal Pharmacopoeia. It also offers detailed information on adulterants, medical parts, photographs, biological classification and their status according to endangered species.

### Current challenges of DNA barcoding in the herbal industry

Nowadays, the usage of plant-based traditional medicines is increasing and the product demand and utility is projected to be 80% of the world’s population who consume the herbal products for wellness and health care. The development of the DNA barcoding technique has been used effectively to identify medicinal plants and herbal products, guaranteeing the safety of consumers. From the careful and comprehensive analysis of literature, it can be ascertained that no one can yet find an efficient barcode for all groups of plants. The limitation of DNA barcoding is to fail the quality of template DNA, the affinity of the primers, the effect of PCR in the herbal products and additives contaminating the DNA samples. In addition, the availability of DNA could be removed or degraded during the manufacturing process, including extensive heat treatment, irradiation, ultraviolet exposure and extractive distillation. DNA is entirely absent in processed products and hence is not suitable for DNA barcoding. Another problem concerning the DNA barcoding technique is that multiple species present in herbal products make PCR biased (Fazekas et al., 2009). The mixtures of herbal drugs can partly overcome PCR bias by doing parallel PCR or cloning specific PCR products into a vector to be able to target the particular species (Newmaster et al., 2013). Another challenge in the field of DNA barcoding is the interference of secondary plant metabolites, including polysaccharides, tannins, alkaloids and polyphenols. These metabolites in plants depend on natural conditions such as seasons, latitude, longitude, and soil fertility, resulting in low-quality DNA and reducing sequencing success. In these cases, metabarcoding and high-throughput sequencing are possible to overcome the issues. In the future, the addition of biological reference material (BRM) and anatomical studies along with sequence data would provide users with high authenticity of medicinal plants and herbal products. Future DNA barcoding perspectives include a novel mini barcode sequence library, BRM library, and anatomical studies for herbal drug authentication.

#### Future perspectives

In recent times, there has been an increase in the demand for environmentally friendly innovations in all sectors of trade. It also impacted the medicinal and health care fields as the global community leaned more towards alternate medicine. The increase in the demand for herbal medicines is used by approximately 80% of the world population for wellness and healthcare, but it is also accompanied by the loss of quality and safety of the products. At present, the DNA barcoding technique is widely used in medicinal plants and has been proven in the authentication of adulterating herbal medicines. Based on the review of literature, single or multiple loci barcode candidates are used to identify and authenticate the medicinal plants since it has not yet identified a universal barcode candidate for all groups of plants. The limitation of DNA barcoding is the quality of the DNA in the herbal products, and many cases contain degraded DNA in the manufactured products. In such cases, minibarcoding technique is worth solving the problem. Another limitation of DNA barcoding is related to multispecies identification from a mixed herbal product; in these cases, metabarcoding, Bar-HRM, and massive sequencing could overcome the limitations. It is clear that a well-established protocol for analytical methods such as organoleptic, microscopic, physiochemical, biochemical and molecular (DNA barcoding) techniques are needed for authentication and quality control of herbal medicines at the industrial level. For instance, coupling DNA barcoding with next-generation sequencing, metabarcoding, and metabolomics will helps to identify each and every marker in the formulation more accurately than all of the traditional methods.

## Conclusion

DNA barcoding-based adulteration detection is still in progress to replace the conventional identification approaches. The chemical analyses are used to detect foreign ingredients and quality control in the herbal drugs, whereas DNA marker-based identification is more beneficial for authenticating the original species. It is essential to add DNA barcoding-based authentication with metabolomics, transcriptomics and proteomics tools to understand adulteration in herbal drugs. This field requires a solid scientific community to add the DNA barcoding protocol in the guidelines to certify the herbal products. Close collaboration between national pharmacopeia agencies and academic or commercial institutes experts in DNA barcoding should be encouraged to pilot DNA barcoding based herbal pharmacovigilance. The routine DNA barcoding authentication could raise the quality and authenticity of the herbal industry along with chemical analytical methods and facilitate pharmacovigilance monitoring and signal detection. In the future, DNA barcoding-based authentication will be allocated in all herbal industries with biomonitoring using available barcodes to detect the adulterants and many DNA barcoding problems will be solved as biological data is progressing rapidly.
